# Supplementary material for: NDR2 Kinase Regulates Microglial Metabolic Adaptation and Inflammatory Response: Critical Role in Glucose-Dependent Functional Plasticity
Source: Int J Mol Sci. 2025 Oct 31;26(21):10630. doi: 10.3390/ijms262110630 (PMC12609617; doi:10.3390/ijms262110630)
Supplement: Supplementary file 1 [file ijms-26-10630-s001.zip › ijms-3803839-supplementary.pdf]

International Journal of
Molecular Sciences

MDPI

Article

NDR2 Kinase Regulates Microglial Metabolic Adaptation and Inflammatory Response: Critical Role in Glucose-Dependent Functional Plasticity

Beatriz Fazendeiro [1,2,3], Ivo Machado [2,4,5], Anabela Rolo [2,4,6], Paulo Rodrigues Santos [1,2,3,7,8,9], António Francisco Ambrósio [1,2,3,10], Paulo F. Santos [1,2,3,6] and Hélène Léger [1,2,3,10,*]

[1] Coimbra Institute for Clinical and Biomedical Research (iCBR), Faculty of Medicine, University of Coimbra, 3000-548 Coimbra, Portugal; beatrizfazendeiro00@gmail.com (B.F.); paulo.santos@fmed.uc.pt (P.R.S.); afambrosio@fmed.uc.pt (A.F.A.); pfsantos@ci.uc.pt (P.F.S.)
[2] Center for Innovative Biomedicine and Biotechnology (CiBB), University of Coimbra, 3004-504 Coimbra, Portugal; imachado@cnc.uc.pt (I.M.); anpiro@ci.uc.pt (A.R.)
[3] Clinical Academic Centre of Coimbra (CACC), 3004-504 Coimbra, Portugal
[4] Center for Neuroscience and Cell Biology (CNC-UC), University of Coimbra, 3004-504 Coimbra, Portugal
[5] Doctoral Program in Experimental Biology and Biomedicine (PDBEB), Institute of Interdisciplinary Research, University of Coimbra, 3004-504 Coimbra, Portugal
[6] Department of Life Sciences, University of Coimbra, 3030-790 Coimbra, Portugal
[7] Laboratory of Immunology and Oncology, Center for Neuroscience and Cell Biology (CNC-UC), University of Coimbra, 3004-504 Coimbra, Portugal
[8] Institute of Immunology, Faculty of Medicine (FMUC), University of Coimbra, 3004-504 Coimbra, Portugal
[9] Center of Investigation in Environment, Genetics and Oncobiology (CIMAGO), Faculty of Medicine, University of Coimbra, 3001-301 Coimbra, Portugal
[10] Association for Innovation and Biomedical Research on Light and Image (AIBILI), 3000-548 Coimbra, Portugal
* Correspondence: hleger@uc.pt or hleger.icbr@gmail.com; Tel.: +351-239-480-287

Supplementary Materials

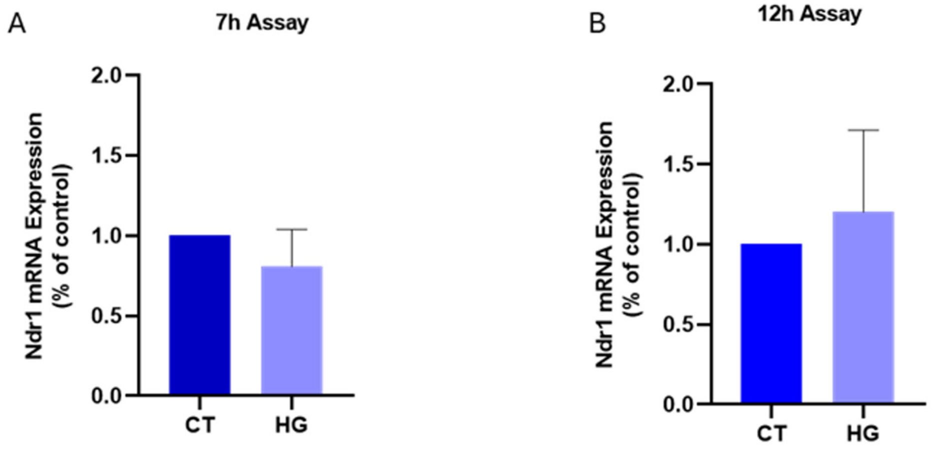

Supplementary Figure S1. High glucose does not affect the Ndr1 mRNA expression in BV-2 microglial cells. Expressed mRNA levels of Ndr1 in BV-2 cells exposed to 5.5 mM glucose (CT) or 30.5 mM glucose (HG) in both the 7h assay and the 12h assay were evaluated by qRT-PCR analysis. The results are normalized to control and are expressed as mean ± SEM. Statistical analysis was assessed with Student's t-test; *n*=3-4 independent experiments.

Off targets	Sequence forward primers	Sequence reverse primers
1:intron:Slco6d1	TCGTCGGCAGCGTCAATTCACCAGCTGGGCTTCA	GTCTCGTGGGCTCGGGGACTCTCTCCTCCTCACGT
1:intergenic:Zfp958-Shcbp1	TCGTCGGCAGCGTCAATTCACCAGCTGGGCTTCA	GTCTCGTGGGCTCGGCCCCGTGCACAAACCAAAAT
4:intergenic:Rnf144b-4930471G24Rik	TCGTCGGCAGCGTCAATTCACCAGCTGGGCTTCA	GTCTCGTGGGCTCGGTGTGGTGTTTCGGAGCAGAA
4:intergenic:Vip-Fbxo5	TCGTCGGCAGCGTCCTCTAGACCCAGCCTTCCCT	GTCTCGTGGGCTCGGGTTGCTAGGGGTAGCCAGTG
4:intergenic:Shank2-Gm14372	Primer3: not found at this Tm	Primer3: not found at this Tm
4:intergenic:Gng4-B3galnt2	TCGTCGGCAGCGTCTGGAGCACTGTGTTGTGGAA	GTCTCGTGGGCTCGGGGAAGGCCCTGGACAATTACT
4:intergenic:Trim52-1700128A07Rik	TCGTCGGCAGCGTCTCAGCCACTTGAACAAATGGC	GTCTCGTGGGCTCGGAAGCAGGTAGGCCAAACCAA
4:intergenic:Naaladl2-Nlgn1	Primer3: not found at this Tm	Primer3: not found at this Tm
4:intergenic:Cd70-Tnfsf14	Primer3: not found at this Tm	Primer3: not found at this Tm
4:intergenic:Grik2-Ascc3	Primer3: not found at this Tm	Primer3: not found at this Tm
4:intergenic:A530006G24Rik-Foxa2	Primer3: not found at this Tm	Primer3: not found at this Tm
4:intergenic:Gpr165-Pgr15l	TCGTCGGCAGCGTCAAGCTGACGTGGTCTGTGAG	GTCTCGTGGGCTCGGTCCACTGATCCCTGCCGATA
4:exon:Stk32a	Primer3: not found at this Tm	Primer3: not found at this Tm
4:intergenic:D330037F02Rik-Gadl1	TCGTCGGCAGCGTCCCTTATTTCCTTCCCCAGGGA	GTCTCGTGGGCTCGGCCTGGCCAATGGTCTCTGTT
4:intergenic:Cul3-1700016L21Rik	Primer3: not found at this Tm	Primer3: not found at this Tm

Supplementary Table S1 – Predicted off-target sites for the CRISPR sgRNA targeting *Ndr2* exon 7.

The table shows the top 15 potential off-target loci identified using CRISPOR v.5.2 (https://crispor.gi.ucsc.edu/ (accessed on 03/09/2025)) with the *Mus musculus* genome assembly GRCm39. Candidate sites are ranked by CRISPOR specificity scores, which account for sequence complementarity, mismatch position, and PAM compatibility. For each predicted off-targets, the gene name and possible primers to amplify 100-150 bp around the genomic location are reported.

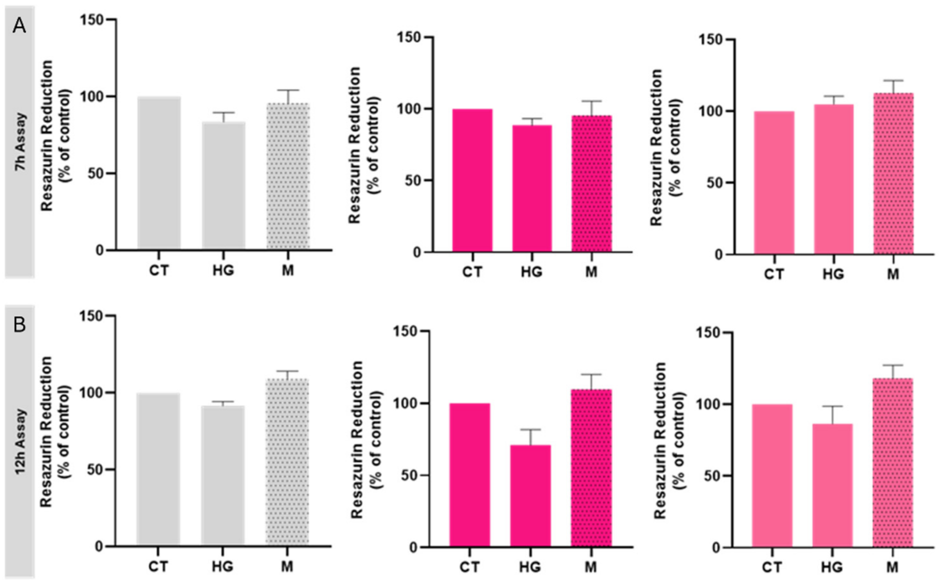

Supplementary Figure S2 – Viability assessment of cultured BV-2 cells exposed to high glucose or mannitol (osmotic control). WT BV-2 (WT) and Ndr2 downregulated BV-2 (Clone 19 and Clone 22) cell cultures were incubated in 5.5 mM glucose (CT), 30.5 mM glucose (HG) or 5.5 mM glucose completed with 25 mM mannitol (M), for different periods: (A) 7h assay; (B) 12h assay. Viability was assessed using the Alamar assay. The results are presented as mean ± SEM. Statistical analysis was assessed with a one-way ANOVA test after confirmation of a Gaussian distribution; * $p \leq 0.05$, compared with WT CT; *n*=6-8 independent experiments.

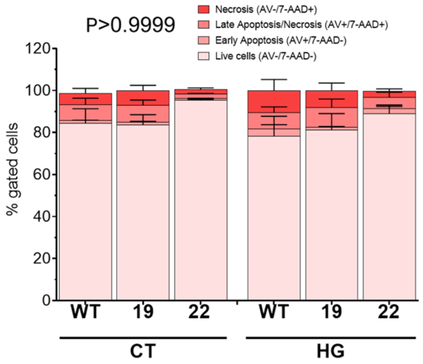

Suppplementary Figure S3 - Determination of apoptotic cell fractions of WT and Ndr2 downregulated BV-2 cells under control and high glucose conditions. Living cells (ANN-V-/7-AAD-), early apoptotic cells (ANN-V+/7-AAD-), late apoptotic/necrotic cells (ANN-V+/7-AAD+) and necrotic cells (ANN-V-/7-AAD+). Asterisks indicate significances compared to control (* $p < 0.05$; ** $p < 0.01$); *n*=3-4 independent experiments.

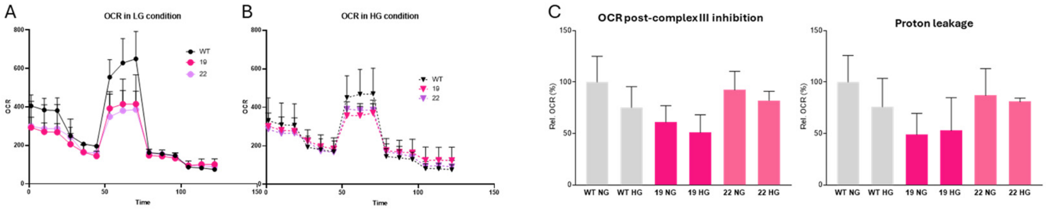

Supplementary Figure S4 - Metabolism assessment of Ndr2 downregulated BV-2 cultured cells. Mitochondrial oxygen consumption of WT and Ndr2 downregulated BV-2 exposed to (A) normal glucose conditions and (B) high glucose conditions. (C) Effect of Ndr2 downregulation on the complex III-linked respiration, measured after injection of rotenone and antimycin A, and on the proton leakage, measured after the injection of oligomycin and before the injection of BAM15, by the Seahorse assay; *n*=3-4 independent experiments.

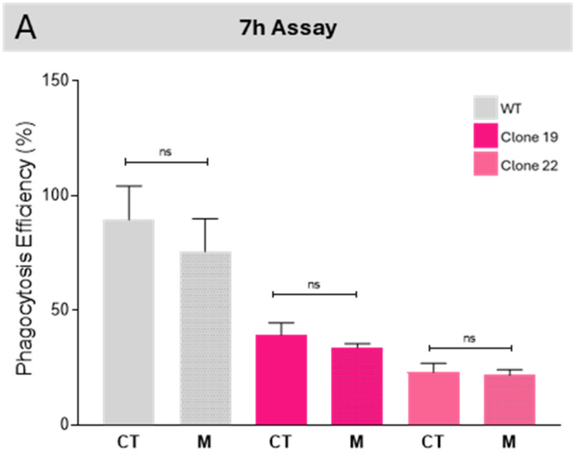

Supplementary Figure S5 - Phagocytic efficiency assessment of cultured BV-2 cells exposed to mannitol (osmotic control). WT BV-2 (WT) and Ndr2 downregulated BV-2 (Clone 19 and Clone 22) cell cultures were incubated in 5.5 mM glucose (CT) and 5.5 mM glucose completed with 25 mM mannitol (M) for 7h assay - as in figure F7. The results are presented as the mean ± SEM, and statistical analysis was assessed with a one-way ANOVA test after confirmation of a Gaussian distribution, compared with their respective CT. (n=3-4).

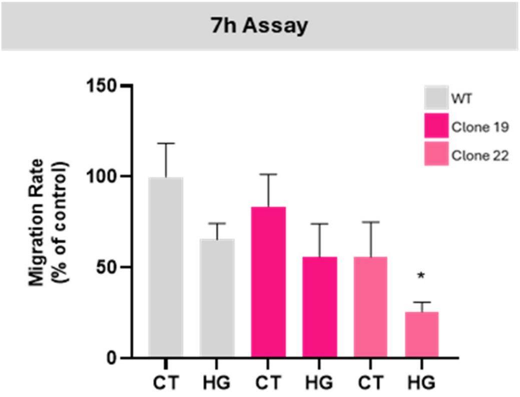

Supplementary Figure S6 - Migration rate of WT and Ndr2 downregulated BV-2 cultured cells exposed or not to high glucose. WT BV-2 (WT) and Ndr2 downregulated BV-2 (Clone 19 and Clone 22) cell cultures were incubated in 5.5 mM glucose (CT) or 30.5 mM glucose (HG) for 7h. The results are presented as the mean ± SEM, and statistical analysis was assessed with a one-way ANOVA test after confirmation of a Gaussian distribution; * $p \leq 0.05$, compared with WT CT; *n*= 4-7 independent experiments.

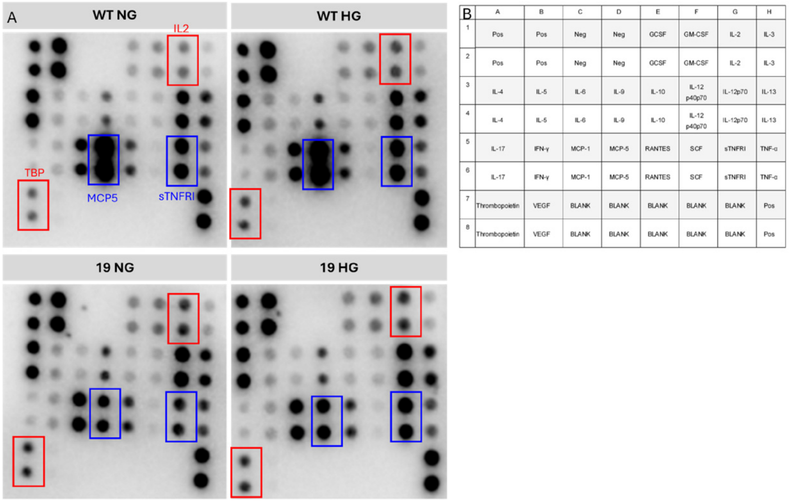

B	A	B	C	D	E	F	G	H
1	Pos	Pos	Neg	Neg	GCSF	GM-CSF	IL-2	IL-3
2	Pos	Pos	Neg	Neg	GCSF	GM-CSF	IL-2	IL-3
3	IL-4	IL-5	IL-6	IL-9	IL-10	IL-12 p40p70	IL-12p70	IL-13
4	IL-4	IL-5	IL-6	IL-9	IL-10	IL-12 p40p70	IL-12p70	IL-13
5	IL-17	IFN-γ	MCP-1	MCP-5	RANTES	SCF	sTNFRI	TNF-α
6	IL-17	IFN-γ	MCP-1	MCP-5	RANTES	SCF	sTNFRI	TNF-α
7	Thrombopoietin	VEGF	BLANK	BLANK	BLANK	BLANK	BLANK	Pos
8	Thrombopoietin	VEGF	BLANK	BLANK	BLANK	BLANK	BLANK	Pos

Suppplementary Figure S7 - Expression of inflammatory factors in WT and Ndr2 downregulated BV-2 under control and high glucose conditions. (A) Images of the cytokine array membranes exposed to the conditioned-media of WT normal glucose (WT NG or CT), WT high glucose (WT HG), and Ndr2 downregulated clone 19 BV-2 cells, exposed to normal or high glucose (19 NG and 19 HG), respectively. (B) Map of the Mouse Cytokine Antibody Array (Membrane, 22 Targets), Abcam reference ab133993.

Abbreviations

The following abbreviations are used in this manuscript:

MDPI	Multidisciplinary Digital Publishing Institute
DOAJ	Directory of open access journals
TLA	Three letter acronym
7-AAD	7-aminoactinomycin D
ADP	adenosine diphosphate
ATP	adenosine triphosphate
AV	annexin V
CT	control
ECAR	extracellular acidification rate
EdU	5-ethynyl-2'-deoxyuridine
GFAP	glial fibrillary acidic protein
HG	high glucose
IBA1	ionized calcium binding adapter protein 1
IL-6	interleukin-6
IL-10	interleukin-10
IL-17a	interleukin-17 a
iPSC	induced pluripotent stem cells
KD	knockdown
NDR	nuclear dbf2-related
NG	normal glucose
NO	nitric oxide
OCR	oxygen consumption rate
qRT-PCR	quantitative reverse transcription PCR
ROS	reactive oxygen species
SDS	sodium dodecyl sulfate
SEM	standard error of the mean
TNF	tumor necrosis factor
WT	wild type
